# Supplementary material for: Novel Virus Air Sampler Based on Electrostatic Precipitation and Air Sampling of SARS-CoV-2
Source: Microorganisms. 2023 Apr 4;11(4):944. doi: 10.3390/microorganisms11040944 (PMC10143856; doi:10.3390/microorganisms11040944)
Supplement: Supplementary file 1 [file microorganisms-11-00944-s001.zip › microorganisms-2267785-supplementary.pdf]

Supplementary materials

Supplementary Method S1. The real-time quantitative PCR (RT-qPCR) for bacteriophage MS2 RNA detection. We used primer-probe sets in the bacteriophage MS2 RNA detection assay (sequences of primer were 5‘-CGTTCACAGGCTTACAAAGTAACCT-3’ and 5’-CCAACAGTCTGGGTTGCCAC-3’, and the sequence of the probe was FAT-AGAATCGCAAATACACCATCAAAGTCGAGGT-TAMRA) [1]. For this primer-probe set, RT-qPCR was performed using One Step Prime Script III RT-qPCR Mix (Takara Bio, Othu, Japan). Twenty microliters of reaction mix comprised 10 μL of enzyme mix, 1 μL of each 0.5 μM primer, 1 μL of 0.5 μM probe, 6.0 μL of RNase Free H2O, and 1 μL of extracted RNA. RT-PCR was performed on a Mygo Mini S Real Time PCR (IT-IS Life Science, Dublin, Ireland) with the following cycle parameters: 5 min at 52 °C for reverse transcription and 10 s of pre-heating at 95 °C followed by 40 cycles of 10 s at 95 °C and 30 s at 60 °C.

Supplementary Method S2. Real-time qPCR (RT-qPCR) for the detection of SARS-CoV-2 RNA. We used a Primer/Probe N2 (2019-nCoV) (Takara Bio, Othu, Japan) in SARS-CoV-2 detection assay targeting the N2 region (sequences of primer were 5’-AAATTTTGGGGACCAGGAAC-3’ and 5’-TGGCAGCTGTGTAGGTCAAC-3’, and sequence of probe was FAM-ATGTCGCGCATTGGCATGGA-BHQ) [2]. For this primer-probe set, RT-qPCR was performed using One Step Prime Script III RT-qPCR Mix (Takara Bio, Othu, Japan). Twenty microliters of the reaction

mix comprised 10 μL of enzyme mix, 4 μL of primer/ probe mix, 1 μL of RNase Free H2O, and 5 μL of extracted RNA. RT-PCR was performed on a Mygo Mini S Real Time PCR (IT-IS Life Science, Dublin, Ireland) with the following cycle parameters: 5 min at 52°C for reverse transcription and 10 s of pre-heating at 95 °C followed by 45 cycles of 10 s at 95°C and 30 s at 60°C. Positive Control RNA Mix (2019-nCov) (Takara Bio, Othu, Japan) was used as positive control.

Supplementary references

1. O'Connell, K.P.; Bucher, J.R.; Anderson, P.E.; Cao, C.J.; Khan, A.S.; Gostomski, M.V.; Valdes, J.J. Real-Time Fluorogenic Reverse Transcription-PCR Assays for Detection of Bacteriophage MS2. *Appl Environ Microbiol* 2006, 72, pp. 478 - 483.
2. Shirato, K.; Nao, N.; Katano, H.; Takayama, I.; Saito, S.;, Kato, F.; Katoh, H.; Sakata, M.; Nakatsu, Y.; Mori, Y.; Kageyama, T.; Matsuyama, S.; Takeda, M. Development of Genetic Diagnostic Methods for Detection for Novel Coronavirus 2019 (nCoV-2019) in Japan. *Jpn J Infect Dis* 2020, 73, pp. 304-307.
